# Amoxicillin-Clavulanate Induced Liver Injury in a Young Female

**DOI:** 10.7759/cureus.33445

**Published:** 2023-01-06

**Authors:** Jennifer Appiah, Ankita Prasad, Viraj Shah, Vraj Patel, Nusha Fareen, Andrea C Marin, Pramil Cheriyath

**Affiliations:** 1 Internal Medicine, Hackensack Meridian Health, Ocean Medical Center, Brick, USA; 2 Internal Medicine, Rajarshee Chhatrapati Shahu Maharaj Government Medical College, Kolhapur, IND

**Keywords:** serum bilirubin, liver enzymes, hepatocellular, alkaline phosphatase, amoxicillin-clavalunate, hepatitis, r score, rucam, liver injury, cholestatic

## Abstract

Amoxicillin-clavulanate (AC) is an antibiotic widely used for various infections. It has rarely been associated with drug-induced liver injury (DILI), mainly in males 55 or older with associated alcohol consumption or medications causing liver injury. Here we present an atypical case of a 22-year-old female with a past medical history of celiac disease and alopecia areata who was prescribed AC in urgent care for bilateral cervical lymphadenopathy, nausea, and chills. Her nausea and vomiting worsened after taking AC for three days, and she developed jaundice. On workup, she was found to have deranged liver functions, and pan-lobular hepatitis was confirmed on liver biopsy. She started to improve symptomatically after withdrawing AC, and her transaminases started showing a decreasing trend.

## Introduction

Amoxicillin is one of the most widely prescribed antibiotics for numerous bacterial infections, including those of the lungs, skin, and soft tissues. Clavulanic acid helps treat bacteria by inhibiting beta-lactamase, the enzyme responsible for most penicillin resistance. The most frequent adverse effects due to the use of amoxicillin-clavulanate (AC) are diarrhea, nausea, pruritus, rash, and vomiting. It is also linked to severe adverse effects, including neutropenia, hemolytic anemia, hepatitis, and Stevens-Johnson syndrome.

Drug-induced liver damage (DILI) is a profound but uncommon side effect of AC. It is a diagnosis of exclusion. Hence, the need to take a thorough history using the Roussel-Uclaf Causality Assessment Method (RUCAM score) to assess the likelihood of DILI once the diagnosis has been suspected [1]. Most incidents occur within the first few days of beginning treatment, although some can even present up to six weeks after initiation [2]. The most common liver damage manifestation is cholestasis; some cases may also be mixed or hepatocellular [3]. The hepatocellular subtype primarily affects younger people, and the cholestatic pattern is more prevalent in elderly patients and those with long-term drug usage [4]. Some studies have implicated older age, longer duration of therapy, and male gender as risk factors for developing DILI [5]. Most AC-induced liver impairment cases are mild to moderate, and symptoms resolve completely. Some patients may develop severe acute liver failure, especially with additional comorbidities or repeated exposures.

## Case presentation

Our patient is a 22-year-old female who presented with jaundice, bilateral cervical lymphadenopathy, and petechial rash one week after returning from a trip to Hawaii. While in Hawaii, she had nausea and chills without any fever with bilateral cervical lymphadenopathy, for which she was prescribed AC (875/125mg tablets twice a day). Her symptoms worsened over the next three days after taking amoxicillin-clavulanate. She developed jaundice, fatigue, loss of appetite, pruritus, and poor appetite, and she presented to the emergency room for further management. She had a past medical history of celiac disease and alopecia areata. She had no history of allergy to medications and had been taking oral contraceptive pills since age 17. Her mother had systemic lupus erythematosus, and her uncle had acute myeloid leukemia. She is a nonsmoker and occasional drinker and has no history of taking recreational drugs. She had been in a monogamous relationship with a male partner for four years. Her physical examination was remarkable for scleral, skin jaundice, and cervical lymphadenopathy; there was no hepatosplenomegaly. The rest of her physical examination was normal.

Initial blood work was significant for leucopenia (White blood count-2000/mm*3(3,400-9,600/mm*3)) with an absolute neutrophil count of (800 cells/mm*3( 2,500-6,000/mm*3), Platelet count (165,000/mm*3(150,000-450,000/mm*3)), Alkaline Phosphatase (ALP) (156 U/L( 20-130 U/L), Aspartate aminotransferase (AST) (249U/L (4-26U/L)), Alanine aminotransferase (ALT) (311U/L (8-33U/L)), Total Bilirubin (3mg/dl( 0.1-1.2mg/dl), Lactate dehydrogenase ( LDH (323IU/L (105-333IU/L) and normal Total protein/ albumin, haptoglobin, Prothrombin time/International Normalized ratio (PT/ INR) and Partial thromboplastin time (PTT). Hepatitis E IgM was negative. Her AC was withheld, and over the next three days, leucopenia with neutropenia resolved with gradual improvement of AST, ALT, and ALP levels over the next eight weeks (Table 1). Total bilirubin continued to increase with the highest of 15.6mg/dl, direct bilirubin at 12.3, and indirect bilirubin at 3.3 mg/dl.

**Table 1:** Liver function tests trend AST: Aspartate Transaminase, ALT: Alanine Transaminase, ALP: Alkaline Phosphatase

	week 9	week 8	week 7	week 6	week 5	week 4
AST	80	78	70	54	46	60
ALT	76	76	65	68	80	118
Total Bilirubin	15.7	17.9	18.7	18	27.2	24.1
ALP	241	255	281	278	340	361
Albumin	2.7	2.7	2.5	2.6	2.5	2.4
Total Protein	6.6	7.3	6.3	7	6.7	6.9

**Figure 1:**
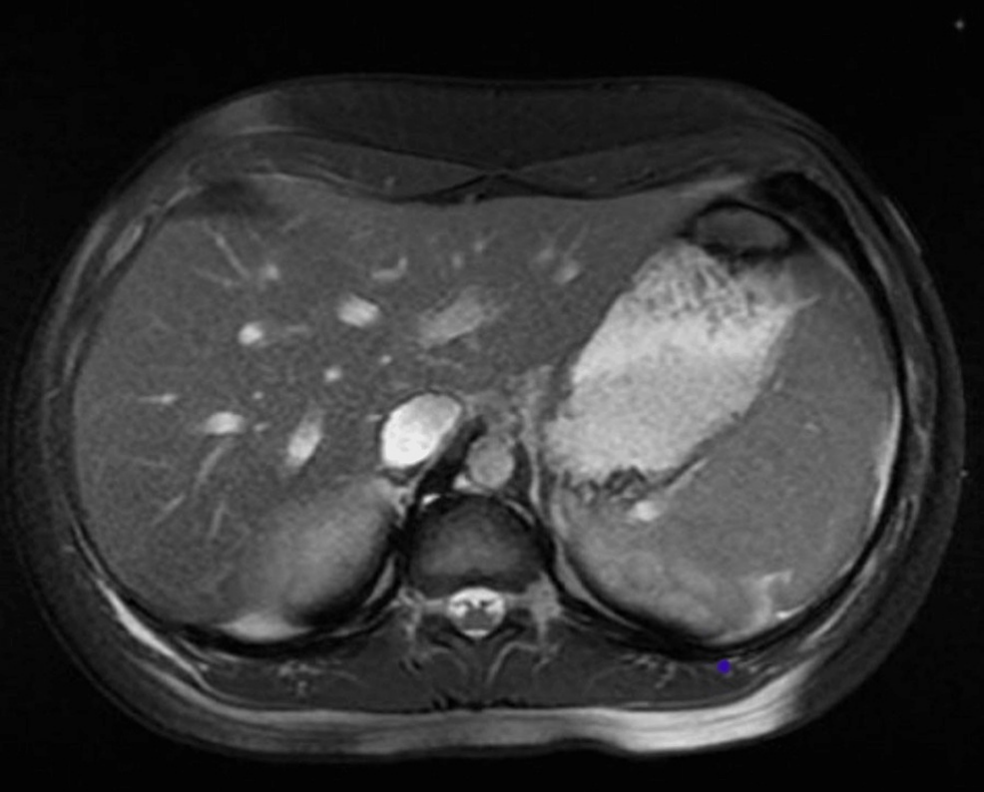
Magnetic resonance imaging (MRI) abdomen (axial) showing normal liver

Extensive workup, including iron panel, Vitamin B12, Folate, Thyroid Stimulating Hormone (TSH), C-Reactive Protein (CRP), Erythrocyte Sedimentation Rate (ESR), Creatinine phosphokinase (CPK), Lipase, HIV, Hepatitis A, B, C, Antinuclear antibody (ANA), Rheumatoid Factor (RF), Ehrlichia antibody, Cytomegalovirus (CMV) IgM, Epstein-Barr virus (EBV) IgM, heterophile antibody screen, Parvovirus B19 Polymerase Chain Reaction (PCR), Leptospirosis IgM, Anti Double-Stranded DNA (dsDNA), Antimitochondrial antibody (AMA) screen, anti-smooth muscle antibody (anti-SMA), Anti neutrophil cytoplasmic antigen (ANCA) Panel, anti-liver-kidney microsomal antibody (anti-LKM antibody), Immunoglobulin A, G, M, Lyme disease antibody with reflex, Serum copper, Ceruloplasmin, anti-Beta-D-Glucan, and Aspergillus antigen were all within normal limits. Computed tomography (CT) scan of the soft tissue of the neck with contrast was significant for shotty scattered jugular chain lymph nodes, and abdominal ultrasound of the liver and spleen were unremarkable. Magnetic resonance imaging of the (MRI) abdomen was unremarkable (Figure 1).

A liver biopsy showed mild nonspecific pan-lobular hepatitis. Her liver enzymes also pointed to a hepatocellular type of DILI. She was prescribed steroids after a liver biopsy which was later tapered and discontinued after diagnosing AC-induced liver injury. The oral contraceptive was discontinued as a precautionary measure. Her liver function tests have since improved, with significant improvement in her symptoms. 

## Discussion

DILI is responsible for roughly 10% of all cases of acute hepatitis, is the source of acute jaundice in 50% of patients with new-onset jaundice, and is responsible for up to 50% of cases of acute liver failure in developed countries [5,6]. Drug-induced liver injury (DILI) due to AC is caused by an immune allergic idiosyncratic reaction in approximately one in 2500 cases [1]. It is commonly associated with human leukocyte antigen (HLA) Class II (DRB1 * 1501-DRB5 * 0101-DQB1 * 0602) and HLA class I (HLA-A*02:02) genes [1]. Numerous genetic variants connected with CYP isoenzymes, HLA alleles, and other drug-processing enzymes have been found and linked to DILI [1,7]. Drug-drug interactions can result in hepatotoxicity. As is the situation with acetaminophen poisoning, it is believed that alcohol use disorder and malnutrition predispose some individuals to DILI.

DILI can be classified based on clinical features into hepatocellular (cytotoxic), cholestatic and mixed injury. Based on the mechanism of injury, it can be classified into predictable and idiosyncratic patterns. Histology can confirm the presence of hepatitis, cholestasis, or steatosis. High serum bilirubin levels and abnormal synthetic function tests are seen in hepatitis. Cholestatic patterns are characterized by increased alkaline phosphatase levels more than twice the normal levels and alanine aminotransferase (ALT) to alkaline phosphatase (ALP) ratio (R-value) of ≤2 along with high serum bilirubin and altered synthetic functions and are considered mixed if the R-value is greater than two but less than five and hepatocellular if the R-value is more than five [8]. The presence of jaundice (serum bilirubin ≥2.5 mg/dL) with elevated serum aminotransferases (>3 times the upper limit of normal) and alkaline phosphatase <2 times the upper limit of normal has been associated with a worse prognosis [9] with a mortality rate as high as 80% if there is an acute liver failure and no transplantation [4,10]. According to Hy's law, with an increase in serum in general, the prognosis for cholestatic injury (i.e., no substantial increase in aminotransferases) is better than that for hepatocellular injury.

Chronic damage is more likely to arise from cholestatic DILI. Generally, chronic injury resolves when the offending substance is discontinued, but this pattern of harm can lead to cirrhosis and, ultimately, liver failure. Cholestasis can take months to resolve and can proceed to chronic liver failure in some cases. Re-exposure to amoxicillin-clavulanate is often associated with a faster onset of severe liver damage than amoxicillin alone. Surprisingly, similar liver damage has not been found with other beta-lactamase inhibitors (tazobactam and sulbactam), but such damage has been recorded with other penicillin combined with clavulanate [1]. Although some clinicians employ corticosteroids to treat severe or prolonged cholestasis, their efficacy is poorly understood, suggesting further study in this area.

Liver biopsy may contribute to diagnostic accuracy, but the histological features of DILI and their relationship to biochemical parameters and outcomes are not well defined. In our patient, the ALT elevation was more than 10 times at presentation, bilirubin thrice the normal, and ALP was much less than twice the normal, all pointing to the liver injury being hepatotoxic rather than cholestatic. Oral contraceptive pills may cause cholestatic hepatotoxicity within the first few cycles following commencement. However, this was less likely to occur in our patient, who has been taking contraceptive pills for around five years.

## Conclusions

This case report aims to raise awareness of the uncommon side effects of a commonly prescribed medication that pose a diagnostic challenge; DILI due to AC is an idiosyncratic reaction to clavulanate and is a diagnosis of exclusion. It is necessary to take a complete medical history, perform a physical examination, and use the RUCAM score to avoid unnecessary diagnostic workup. In most cases stopping the medication improves the patient's symptoms and lowers liver enzymes. However, some cases may proceed to liver failure and require liver transplantation.
